# Measuring Digital Stress in Children: Construct Validity, Model Comparisons, and Measurement Invariance of a Multidimensional Scale (DSS-CH)

**DOI:** 10.3390/children13030405

**Published:** 2026-03-14

**Authors:** Arvid Nagel, Felix Kruse

**Affiliations:** 1 Media Effects Lab, University of Teacher Education NMS Bern, Waisenhausplatz 29, 3011 Bern, Switzerland; 2 Institute of Physical Education, Sports and Health, St. Gallen University of Teacher Education, Notkerstrasse 27, 9000 St. Gallen, Switzerland

**Keywords:** digital stress, children’s digital media use, multidimensional construct, measurement instrument, measurement model comparison, psychometric validation

## Abstract

**Background:** The use of digital media in childhood offers both opportunities and risks. Digital stressors—such as excessive screen time, constant availability, information overload, and social media pressures—affect primary school children but have been rarely studied systematically. Despite growing research, no validated instruments adequately capture how younger children perceive and express digital stress. This study presents the development and validation of a three-dimensional instrument for children under 14: the “Digital Stress in Children” scale (DSS-CH). The DSS-CH is theory-driven and child-appropriate, with three interrelated but distinct dimensions: (1) excessive screen time, (2) compulsive media behavior, and (3) approval anxiety. **Methods:** In a cross-sectional survey of *n* = 907 Swiss primary school children (grades 5–6; ages 10–14), participants completed an online questionnaire in class. Latent variable modeling with cluster-robust inference accounted for classroom nesting. Competing models (1-, 2-, 3-factor CFA; ESEM; bifactor-ESEM) were evaluated. **Results:** The 1-factor CFA fit poorly (CFI ≈ 0.81; RMSEA ≈ 0.15), while the 3-factor CFA showed acceptable fit (CFI ≈ 0.96; RMSEA ≈ 0.07). Allowing cross-loadings improved fit substantially in the 3-factor ESEM and bifactor-ESEM (CFI ≈ 0.999; RMSEA ≈ 0.01), supporting a general digital stress factor alongside facet-specific variance. Subscales showed good reliability (ordinal α ≈ 0.81 − 0.89) and moderate intercorrelations (r ≈ 0.28 − 0.47). Scalar invariance across gender and age was supported (ΔCFI ≤ 0.003; ΔRMSEA ≤ 0.012). **Conclusions:** The DSS-CH demonstrates good reliability, model fit, and measurement invariance. It provides valid evidence for interpreting children’s digital stress as three related facets and can help identify elevated stress profiles to inform preventive efforts.

## 1. Introduction

In recent years, children and adolescents have experienced significant changes in their daily lives, largely due to the rapid integration of digital technologies. Prensky famously described this generation as “Digital Natives,” highlighting the profound impact of technology on learning, socialization, and leisure [1]. Digital media enable people to stay constantly online while maintaining connections at all times [2,3,4]. The media usage of elementary school children has evolved considerably, with increasing access to smartphones, tablets, and computers, as well as a wide range of interactive digital content. While traditional media such as television and books remain relevant, digital platforms have become central to children’s daily routines, shaping both leisure activities and social interactions with peers and family [5,6,7]. Internet use has become an integral part of everyday life for many children, starting at increasingly younger ages. According to the 2024 KIM study by the Media Education Research Association Southwest—a recent German survey on children’s media access—more than half of Internet-using children aged 6–13 go online daily.

Intensive engagement with digital offerings now extends into elementary school, often including social media use, despite official age restrictions typically permitting access only from age 13 [5]. Consequently, the childhood experience of contemporary elementary school children differs markedly from that of children around the turn of the millennium. The age at which they first access the Internet, acquire a mobile phone, and begin using social media has gradually shifted from early adolescence to childhood over recent years [5]. The study also indicates that smartphones are present in nearly all households (98%) and are the device most commonly owned by children themselves (46%), with ownership rising sharply with age—from 11% among 6–7-year-olds to 79% among 12–13-year-olds [5]. Children basically use smartphones for communication, social media, gaming, entertainment, and increasingly for educational and creative purposes, with usage varying by age [8]. 

The increasing integration of digital technologies into everyday life presents both opportunities and challenges. While digital media facilitate learning, communication, and entertainment, their pervasive use also introduces stressors for children, such as information overload, constant connectivity, and social pressures. These experiences, collectively referred to as digital stress, can already affect well-being and development in early childhood [9]. Although research on digital stress (“technostress”) is well-established among adolescents and adults, empirical evidence on younger children, particularly primary school students, remains limited. Studies indicate that even elementary school children experience situations in which they feel overwhelmed, distracted, or socially pressured by digital media, and that digital stressors—such as technical problems, information overload, and social comparisons—are measurably associated with psychological well-being and development [10,11].

The COVID-19 pandemic has further amplified digital stress, as social distancing and school closures shifted many interactions to online platforms such as WhatsApp, Snapchat, and Instagram [12,13]. While this transition was necessary to maintain social contact, it may have increased pressure through constant availability, multitasking, and fear of missing out. In this context, the present study aims to address a significant research gap by introducing a novel instrument for assessing digital stress in primary school children.

### 1.1. Understanding Digital Stress: Theoretical Frameworks and Empirical Insights

Digital stress can be understood within the broader theoretical framework of stress research, based on the transactional model of stress developed by Lazarus and Folkman, which emphasizes the subjective perception of demands relative to available coping resources [14]. Within this framework, stress arises when individuals perceive that the demands placed upon them exceed their coping capacity. Building on this theoretical foundation, digital stress has increasingly attracted scholarly attention and has been conceptualized across a range of empirical investigations [15,16,17,18,19,20,21,22,23]. 

Much of this research is theoretically grounded in Brod’s [24] seminal framework of technostress (Consistent with early conceptualizations of “technostress,” the literature continues to use a range of terms for similar or overlapping constructs related to digital stress, such as Facebook-Induced Stress [25], Social Pressure and Communication Load [18], Facebook Related Stress [26], Connection Overload [27], Social Media Fatigue [28], Accessibility Stress/Availability Demands [29], and Mobile Entrapment [30]. In contemporary research, the term “technostress” is commonly applied to stress associated with workplace ICT use, whereas stress resulting from ICT use in personal contexts is usually labeled as “digital stress” [31]), which delineates the perceived incapacity of individuals to adequately cope with technological demands in a manner conducive to psychological well-being and somatic health. Whereas technostress traditionally refers to the maladaptive interaction between individuals and technological systems, the notion of digital stress represents a conceptual expansion that integrates additional dimensions of strain and psychosocial burden. Digital stress is not restricted to the immediate cognitive load or behavioral demands associated with active media engagement; it also encompasses stressors arising from the temporary deprivation of digital affordances as well as from vicarious usage by third parties [32].

This reconceptualization broadens the scope of analysis to include both direct and indirect stress-inducing mechanisms. On the one hand, digital stress captures task-related stressors such as information overload, multitasking requirements, and interruptions that undermine attentional control and executive functioning. On the other hand, it incorporates socio-emotional stressors embedded in digitally mediated environments, for instance, the fear of missing out (FoMO), the anticipatory anxiety associated with permanent reachability, and the erosion of boundary management between professional and private life [9]. Digital stress has been defined as “stress resulting from a strong and perhaps nearly constant use of information and communication technology (ICT), triggered by permanent access to an overwhelming amount and diversity of (social) content” [17] (p. 237). Steele et al. [20] (p. 16) further describe it as “the stress and anxiety that accompany notifications from and the use of information and communication technologies via mobile and social media” and conceptualize it in a multidimensional model comprising four key facets: Availability Stress (stress from expectations of constant digital accessibility), Approval Anxiety (fear of negative evaluation of one’s digital presence), FoMO (stress from exclusion from rewarding social experiences), and Connection Overload (subjective overload from incoming digital stimuli). 

This framework highlights that digital stress is shaped by individuals’ personal experiences and coping resources, rather than being determined solely by the quantity of media use. People can respond very differently to the same digital stimuli: for example, a certain number of smartphone notifications may be easily manageable for some, while causing significant digital stress for others [17]. By focusing on subjective perception rather than usage metrics, this approach clearly distinguishes digital stress from mere social media engagement or screen time [20]. 

Building on earlier research, Hall et al. [16] validated four stress factors and, through exploratory factor analysis, introduced a fifth, resulting in a five-dimensional digital stress scale with 24 items [33]. This scale has been employed and validated in several follow-up studies [2,34], and Xie et al. [35] further adapted it into Chinese, empirically confirming the five-dimensional structure and demonstrating its reliability and validity among young adults. Research on digital stress has predominantly examined the use of information and communication technologies (ICT) in professional settings, e.g., [36,37] or in private life [18,21]. These studies identify various stressors, their implications for health and well-being, and the coping mechanisms individuals employ [31]. 

As highlighted by Steele et al. [20], there is growing evidence that digital stress is an important factor in understanding the relationship between digital media use and psychosocial outcomes in adolescents and young adults [18,27,30]. Digital stress may negatively affect mental health. Most research on this topic has focused on adult participants or on mixed samples including both adolescents and adults [2]). Research highlights four main dimensions of digital stress that shape how mobile and social media use affect mental health. Constant availability predicts higher stress and depressive symptoms [29], while social pressures to remain reachable contribute to feeling overwhelmed [18]. FoMO links adolescents’ social media use to parent-reported anxiety and depression [38,39]. High communication load increases perceived stress and is indirectly associated with burnout, depressive symptoms, and anxiety [18]. Approval anxiety, observed in a sample of 164 U.S. female university students, arises when users feel compelled to present themselves positively online and is linked to higher stress, more depressive symptoms, and lower resilience, especially among those whose self-worth depends on social media feedback [40]. 

According to Hall et al. [16], the relationship between social media use and psychological functioning is mediated by approval anxiety and FoMO, but only in adolescent populations. Among 12- to 16-year-olds in a rural, lower-middle-class area of the southeastern U.S., 42.5% reported experiencing digital stress at least sometimes. No racial or ethnic differences were found, but girls reported slightly higher stress levels than boys [41]. A meta-analysis by Khetawat and Steele [42] indicates that digital stressors—including availability stress, approval anxiety, FoMO, connection overload, and online vigilance—exhibit moderate and comparable associations with psychosocial stress, with age moderating the relationship only for connection overload. 

Accordingly, digital stress can be theorized as a multidimensional psychosocial construct situated at the intersection of stress research, media psychology, and health sciences. It encompasses both primary appraisal processes (e.g., the subjective evaluation of digital demands as threatening or overwhelming) and secondary appraisal mechanisms (e.g., the perceived insufficiency of coping resources), thereby linking classical stress theory with contemporary analyses of digital media ecologies [14]. Given this subjectivity, the use of nuanced measurement instruments is necessary to accurately capture individuals’ perceptions and responses to digital stressors [43,44]. Importantly, digital stress cannot always be clearly distinguished from general, non-digital stress experienced by individuals; however, the ubiquitous nature of digital technologies and the demands they impose substantially contribute to heightened subjective stress levels, underlining the critical role of digitalization in shaping contemporary stress experiences [9,16].

### 1.2. Conceptualizing and Measuring Digital Stress in Children (DSS-CH): A Multidimensional Approach

Despite the increasing use of digital technologies among children, there is currently no validated, multidimensional instrument for assessing digital stress in children under 14 years of age. Existing measures, such as the Digital Stress Scale (DSS), were developed and validated primarily for adolescents aged 14 years and older, as well as young adults [16]. The DSS evaluates digital stress across five dimensions—availability, social validation anxiety, fear of missing out (FoMO), connection overload, and online vigilance—and has been successfully applied in studies with adolescents and young adults. However, no adaptation exists for younger children, leaving the applicability of these scales to this age group uncertain. Age-appropriate instruments are critical for accurately capturing digital stress in children, as existing tools do not account for developmental differences in digital media use and stress perception. Without such measures, the impact of digital technology on the well-being and mental health of younger children cannot be systematically investigated. Developing and validating multidimensional scales for this population is therefore a pressing research priority.

For children under 14 years, compared with mid- to late-adolescents and young adults, peer ecologies tend to be smaller and more supervised, digital autonomy and boundary management are more externally regulated (by parents/teachers), and social-cognitive operations that underpin strategic online self-presentation (e.g., reflected appraisal, audience awareness, fine-grained social comparison) are still consolidating [44]. Consequently, evaluative pressures around visibility and feedback may be experienced more globally (as a diffuse worry about peers’ reactions), and compulsive and attention demands from connectivity and notifications may be perceived as a more undifferentiated sense of being driven by an urge and being overrun, rather than as analytically distinct subcomponents such as availability stress, connection overload, and online vigilance that are often separable in older samples [3,16,20].

In contrast to adolescents, for whom differentiated dimensions such as availability stress, connection overload, and online vigilance have repeatedly emerged as separable constructs, children aged 10–14 may not yet cognitively or experientially differentiate these demands to the same extent. At this developmental stage, digital stressors are more likely to be appraised in a global and experience-near manner, reflecting a general sense of pressure, urgency, or loss of control rather than analytically distinct subcomponents. This developmental compression of digital demands is consistent with research indicating that younger respondents tend to exhibit broader latent structures and less differentiated factor solutions. Accordingly, our conceptualization prioritizes developmental fidelity over strict replication of adolescent taxonomies, while remaining theoretically anchored in established digital stress frameworks.

General psychometric work supports in this context that younger respondents tend to represent constructs less differentially, yielding broader factors and higher interfactor correlations, so that finer distinctions observable in older samples often have not yet crystallized [45,46]. This developmental picture suggests that a streamlined representation may capture children’s digital stress with greater fidelity and stability. A measurement-economy perspective complements this rationale. In adolescent/young-adult research, canonical digital-stress facets (approval anxiety, fear of missing out, availability/connection overload, vigilance) typically show similar, medium-sized associations with psychosocial distress, while incremental evidence is scarce and age moderates only selected links [42]. When facets are highly collinear at the construct level, enforcing fine-grained factor distinctions in younger children may add complexity without clear incremental validity and can increase the risk of unstable loadings or ill-conditioned models. A brief, developmentally tuned instrument that aggregates closely related compulsive processes into a single, comprehensible factor (availability, overload, vigilance), retains a clear interpersonal factor (approval anxiety), and captures time/self-regulation strain (excessive screen time) therefore seems a prudent starting point—one that is widely compatible with, but not constrained by, adolescent taxonomies [16,20]. Two construct decisions deserve explicit justification here. First, we conceptualize “excessive screen time” as an appraisal-based stress facet. We emphasize that EST reflects children’s subjective perception of stress and loss of control, rather than objective screen time. The wording of our items (e.g., “I spend more time on digital devices than I would like”) indexes self-discrepancy and perceived loss of control, consistent with transactional definitions of stress as a subjective evaluation of demands relative to goals and coping resources [14]. In pre- and early adolescent contexts, where routines and boundaries are often externally set, such perceived overuse may be a developmentally salient, readily reportable source of strain that is psychometrically distinct from availability/information load and approval anxiety, while remaining compatible with broader frameworks that treat digital stress as appraisal rather than exposure [3,20]. This also distinguishes EST from constructs such as problematic use or self-regulation, which focus on behavioral frequency or regulatory skills rather than perceived strain.

Second, FoMO is not included as a core subscale. Although prominent in adolescent models, FoMO is typically defined as a domain-general concern about missing rewarding experiences that may occur offline or online. Many validated items contain no inherent reference to digital affordances [16,39]. Including FoMO would therefore dilute construct specificity for a digital stress measure. In addition, for child samples, instrument brevity and age-appropriate wording are prioritized to reduce cognitive load, satisficing, and missingness in classroom administration [45,46]. Excluding FoMO helps maintain a concise, developmentally tuned scale that targets digital-affordance–anchored stressors while still allowing FoMO to be analyzed as an external correlate in convergent-validity tests and, in future work, to be reconsidered as a separable facet if it shows distinct predictive value as public self-presentation and platform-based feedback gain salience in early to mid-adolescence [20,41].

Following this rationale, the Digital Stress Scale for Children (DSS-CH) serves as a brief, parsimonious, and practically applicable measure for children and early adolescents, comprising three dimensions:

(1) *Excessive screen time (EST):* This dimension reflects stress arising from prolonged or uncontrolled use of digital devices, including computers, tablets, smartphones, or gaming consoles. Children may experience fatigue, irritability, reduced attention, or sleep disturbances as a result of overexposure to screens, highlighting the cumulative burden of continuous digital engagement on their mental and physical well-being.
(2) *Compulsive media behavior (CMB):* This dimension captures the stress associated with the pressure of being continuously reachable and exposed to a high volume of digital information. Children may feel compelled to respond immediately to messages, notifications, or online demands, leading to feelings of being overwhelmed, distracted, or cognitively overloaded. The incessant flow of information can limit their ability to focus, plan, or regulate emotions effectively, contributing to overall digital stress.
(3) *Approval anxiety (AAN):* This dimension pertains to stress arising from interactions on social media platforms, including social comparisons, peer evaluation, and perceived social expectations. Children may experience anxiety, fear of missing out (FoMO), or negative self-evaluation when engaging with peers online. The constant monitoring of likes, comments, or shared content can intensify emotional responses and create social pressures that are uniquely amplified in digital contexts.

## 2. The Present Study

Previous scales assessing digital stress were primarily developed for adolescents and adults. While questionnaires on media use exist for children, no validated instruments systematically capture digital stressors in this age group, representing a notable research gap. The present study aims to address this gap by developing a psychometrically sound, age-appropriate scale for primary school students in grades 3 to 6 (approximately 7–14 years). Items were formulated in simple, child-friendly language to enhance comprehension and response accuracy. The item pool was generated based on theoretical models and five qualitative interviews with children, reviewed by four experts in education and psychology, and tested in a pilot study with 240 students from grades 3 to 6 from three primary schools. Initial items were derived from recurring themes identified in the qualitative interviews, particularly situations in which children described digital media use as stressful or difficult to regulate. These statements were translated into concise, age-appropriate item wording and iteratively refined to ensure clarity and relevance for the target age group. The preliminary item pool was subsequently reviewed by experts to evaluate content validity and remove redundant or ambiguous items. In the pilot study, items were further examined for comprehensibility and response variability, and the final set of 10 items was selected based on conceptual coverage and psychometric considerations.

Motivated by the pressing need for a validated, child-appropriate measure of digital stress, this study addresses the following research questions:

(1) Which latent structure best represents children’s digital stress as assessed by the DSS-CH items? Competing representations are evaluated, including (a) ICM-CFA solutions (1-, 2-, and 3-factor), (b) ESEM models that allow theoretically plausible cross-loadings, and (c) bifactor-ESEM models testing whether item covariation is driven by both a general digital stress factor and residual facet-specific variance.
(2) Do the DSS-CH subscale scores show adequate internal consistency and model-based reliability, and are the facets sufficiently distinguishable to justify reporting separate subscale scores? We evaluate internal consistency (ordinal α) and model-based reliability indices (e.g., CR/AVE) alongside interfactor correlations.
(3) Is the DSS-CH measurement model invariant across key demographic groups (gender and age group), such that group comparisons of latent means and associations are interpretable? We test configural and scalar invariance (loadings and thresholds) for categorical indicators using multi-group models and changes in approximate fit.

## 3. Methods

### 3.1. Participants and Procedure

Participants were primary school students (Grades 5–6) from the German-speaking region of Switzerland, recruited via school principals. Participation required parental consent and child/adolescent assent. The study protocol was approved by the ethics committee of the [Name of the University]. No incentives were provided. The analytic sample for descriptive statistics comprised *n* = 907 students (girls: *n* = 444, boys: *n* = 463) with at least one valid response on the digital stress items. Students were nested in 82 classrooms (mean cluster size $\overset{-}{k}$ ≈ 10.93), motivating cluster-robust inference. Age ranged from 10 to 14 years (M = 11.43, SD = 0.74). For developmental invariance testing, students were grouped into 10–11 years (*n* = 494) vs. 12–14 years (*n* = 410), reflecting a transition from late childhood into early adolescence with meaningful shifts in autonomy, peer orientation, and social-evaluative concerns. The survey was administered during regular class time using an online questionnaire. Data collection was supervised by trained teachers following standardized instructions, and students completed the survey individually, with assistance available for procedural questions. Age and gender were assessed using self-report items at the beginning of the questionnaire.

### 3.2. Measures

Digital stress was operationalized using 10 self-constructed items with a 4-point response format (1 = not true at all, 2 = mostly not true, 3 = mostly true, 4 = totally true). Based on this rationale, three dimensions were specified:

(a) Excessive Screen Time (3 items) (e.g., “spending more time on digital devices than intended”)
(b) Compulsive Media Behavior (3 items) (e.g., “feeling restless when unable to access the smartphone/computer”)
(c) Approval Anxiety (4 items) (e.g., “worry that posts will not be well received/not enough likes”).

### 3.3. Analytic Strategy

The statistical analyses were performed in three steps:

(1) Data screening, descriptives, reliability and clustering: We inspected response distributions (range compliance 1–4), item missingness, and computed scale means. Internal consistency was estimated using Cronbach’s α and ordinal α computed from polychoric correlations. We also report composite reliability (CR) and average variance extracted (AVE) from an ordinal CFA model in lavaan. To quantify classroom dependence, we computed ICC(1) for each scale mean. These ICCs informed the decision to use cluster-robust modeling in Mplus.
(2) Factor structure models: Because indicators are ordinal, all latent variable models were estimated in Mplus 8.11 using the WLSMV estimator with THETA parameterization and TYPE = COMPLEX to obtain robust standard errors and chi-square tests under classroom clustering. Model fit was evaluated using CFI, TLI, RMSEA (with 90% CI), and SRMR, interpreted using common guidelines [47]. We compared a sequence of increasingly flexible structural representations:
  - ICM-CFA models (independent clusters) to test whether the construct is best represented as one, two, or three correlated factors.
  - ESEM with target rotation to relax the unrealistic “zero cross-loading” constraint inherent to ICM-CFA.
  - Bifactor-ESEM to test the substantive hypothesis that item covariance is partly driven by a general digital stress factor (G) while retaining specific factors that capture residualized domain-specific variance (EST, CMB, AAN). This directly addresses the theoretical expectation of an overarching stress liability with overlapping facets, and it provides a sharper diagnostic of whether “multidimensionality” reflects correlated facets, a dominant general factor, or both.
(3) Measurement invariance: We evaluated measurement invariance across age group (10–11 vs. 12–14) and gender (girls vs. boys) using multi-group categorical CFA models under WLSMV/THETA/COMPLEX, to align with common practice in the measurement invariance literature. Because Mplus does not allow MODEL = METRIC for categorical outcomes, invariance was tested by explicit equality constraints on loadings (metric) and thresholds (threshold/scalar). Model comparisons were based on changes in approximate fit (ΔCFI, ΔRMSEA) using Chen’s [48] recommendations. Because Mplus does not allow MODEL = METRIC for categorical outcomes, invariance was tested by comparing a configural model (all parameters free) against a scalar model (loadings and thresholds constrained to equality across groups). This step-up approach is standard for categorical indicators, where metric and scalar invariance are typically evaluated simultaneously to ensure the comparability of latent scores. Model comparisons were based on the DIFFTEST procedure for χ^2^ differences and changes in approximate fit indices (ΔCFI ≤ 0.010; ΔRMSEA ≤ 0.015) according to Chen [48].

## 4. Results

### 4.1. Preliminary Analyses: MISSINGNESS, Distributions, and Clustering

Item-level missingness was very low (< 1.% per item). Seven participants had missingness on all digital stress items and were excluded from item-based analyses. Given the small amount of missing data, analyses proceeded using all available responses. Item distributions showed moderate skew for EST items, stronger skew for CMB, and pronounced floor effects for AAN items, which is typical for social-evaluative anxiety content in younger samples and supports the choice of ordinal modeling. Classroom clustering was non-trivial for scale means (ICC(1) = 0.089−0.164), indicating that cluster-robust inference was appropriate. Accordingly, Mplus factor models used TYPE = COMPLEX with classroom as the clustering variable.

### 4.2. Descriptives, Reliability, and Associations Among Subscales

Descriptive statistics and reliability estimates are presented in Table 1. Ordinal alpha indicated good internal consistency for all three subscales (α_ordinal = 0.81–0.89). Model-based reliability was also satisfactory (CR = 0.83–0.90), with AVE values supporting convergent validity (AVE = 0.59–0.70). Subscales were moderately correlated (Pearson r = 0.28–0.47, all ps < 0.001, Table 2), consistent with partially distinct but meaningfully overlapping facets of digital stress.

### 4.3. Factor Structure: CFA vs. ESEM vs. Bifactor-ESEM

The one-factor CFA fit the data poorly, indicating that digital stress could not be adequately represented as strictly unidimensional. Two-factor CFA solutions improved fit but remained below commonly used benchmarks. The three-factor CFA showed acceptable fit (RMSEA = 0.068, CFI = 0.965, SRMR = 0.047), supporting the theorized three-facet structure. Consistent with modern factor-analytic guidance, we then tested whether the strict independent-clusters CFA assumptions were unnecessarily restrictive. The 3-factor ESEM yielded excellent fit (CFI = 0.999; RMSEA = 0.013; SRMR = 0.012) and reduced factor correlations (Table 3), indicating that allowing small cross-loadings provided a more realistic representation of the data. The bifactor-ESEM also showed excellent fit (CFI = 0.999; RMSEA = 0.012; SRMR = 0.012), supporting a model in which item covariance reflects both a broad general digital stress factor and residual facet-specific variance. Table 4 summarizes model fit.

### 4.4. Loading Pattern

In the ESEM, the pattern of loadings largely supported the intended three-facet structure. EST items showed strong primary loadings on EST (λ = 0.76–0.86) with only trivial cross-loadings on the other facets. For CMB, two indicators displayed clear primary loadings on the intended factor (VAR17_5 “always reachable”: λ = 0.66; VAR17_6 “reply immediately”: λ = 0.86), accompanied by small secondary loadings (e.g., VAR17_5 on EST: 0.20; on AAN: 0.13). In contrast, VAR17_2 (“restless without device”), although intended as CMB, loaded most strongly on EST (λ = 0.48), with comparatively smaller loadings on CMB (0.20) and AAN (0.17), suggesting that this item may reflect loss-of-control/overuse aspects rather than domain-specific compulsive features in this age group. AAN items showed very high primary loadings on AAN (λ = 0.71–0.94) and generally small cross-loadings; one item (VAR19_10) exhibited a modest secondary loading on EST (0.17) while retaining a clear primary loading on AAN (0.71).

In the bifactor-ESEM (B-ESEM), the general factor (G) loaded consistently across all items (λ = 0.50–0.71), supporting an overarching digital stress dimension. At the same time, several specific factors retained meaningful variance beyond G. The EST-specific factor remained substantive for the three EST items (λ = 0.49–0.71). For CMB, the specific factor was strongly defined by “always reachable” (λ ≈ 0.45) and “reply immediately” (λ ≈ 0.81), whereas VAR17_2 showed no meaningful loading on the CMB-specific factor, indicating that this indicator primarily contributes to the general digital stress factor rather than to residualized compulsive-media variance. The AAN-specific factor also remained strong (λ ≈ 0.46–0.75), indicating facet-specific social-evaluative strain beyond the general factor. Overall, the loading pattern is consistent with an interpretation in which digital stress reflects both a broad common liability and distinguishable components related to overuse/loss of control (EST), compulsive–availability pressure (CMB), and social-evaluative concerns (AAN). Note that it is not necessary for all S-factors to be well-defined, as these factors can account for residual specificities shared among subsets of items [43]. Table 5 reports the standardized (STDYX) loadings.

### 4.5. Measurement Invariance Across Age and Gender

For gender, the configural model showed a good fit to the data (χ^2^ = 191.98, df = 64, CFI = 0.973, RMSEA = 0.066), indicating that the basic factor structure was equivalent for girls and boys (see Table 6). When constraining factor loadings and item thresholds to equality (scalar invariance), the model fit remained equally (χ^2^ = 202.25, df = 88, CFI = 0.976, RMSEA = 0.054). The χ^2^ difference test was non-significant (Δχ^2^ = 19.97, df = 24, *p* = 0.698), and the changes in fit indices (ΔCFI = +0.003, ΔRMSEA = −0.012) were well within the recommended thresholds. These results provide strong evidence for full scalar invariance across gender. Regarding age, the configural model comparing students aged 10–11 with those aged 12–14 also demonstrated good fit (χ^2^ = 208.09, df = 64, CFI = 0.967, RMSEA = 0.071). The more restrictive scalar model showed an equally robust fit (χ^2^ = 232.32, df = 88, CFI = 0.967, RMSEA = 0.060). The DIFFTEST procedure indicated no significant loss of fit (Δ χ^2^ = 33.69, df = 24, *p* = 0.090), supported by stable approximate fit indices (ΔCFI = 0.000, ΔRMSEA = −0.011). Thus, scalar invariance was also established for the age groups. In sum, the results confirm that the digital stress dimensions are measured consistently across these demographic subgroups, allowing for valid comparisons of latent means and associations.

Given scalar invariance across gender and age group, we estimated latent mean differences within the scalar-invariant models (reference group fixed to 0). Older children (12–14 vs. 10–11) showed higher latent means on EST (ΔM = 0.255, *p* = 0.020; Δ ≈ 0.16 SD), CMB (ΔM = 0.363, *p* = 0.001; Δ ≈ 0.31 SD), and AAN (ΔM = 0.746, *p* = 0.006; Δ ≈ 0.41 SD). For gender (boys vs. girls; girls as reference), boys showed lower latent means on EST (ΔM = −0.248, *p* = 0.030; Δ ≈ −0.16 SD) and AAN (ΔM = −0.794, *p* = 0.031; Δ ≈ −0.39 SD), whereas the difference on CMB was not statistically significant (ΔM = −0.136, *p* = 0.234; Δ ≈ −0.11 SD).

## 5. Discussion

The present study aimed to advance measurement work on digital stress in late childhood and early adolescence by developing and psychometrically evaluating a brief, developmentally appropriate instrument (DSS-CH). This effort must be interpreted within the broader state of the field: despite increasing scholarly attention, digital stress still lacks a widely accepted and clearly delimited conceptualization, and the empirical evidence linking digital technology use to mental health remains mixed, often small in magnitude, and rarely suited for causal inference or concrete recommendations [49,50,51]. In this context, rigorous and developmentally sensitive measurement constitutes a necessary—though not sufficient—precondition for cumulative theory testing.

Recent integrative accounts argue that inconsistent findings in digital media research are partly attributable to insufficient construct specificity and to treating “social media use” or “screen time” as monolithic exposures rather than focusing on underlying mechanisms and affordances (e.g., quantifiability of feedback, public visibility, constant availability, variability of social rewards) that may interact with developmental vulnerabilities [51]. This critique applies directly to digital stress research. The construct has frequently served as an umbrella term for heterogeneous experiences—communication overload, permanent reachability, evaluation anxiety, FoMO, online vigilance, and technostress—often with partially overlapping terminology [17,18,20]. Consequently, the empirical base remains dominated by cross-sectional associations, with limited longitudinal and very limited causal evidence, even in adolescent and adult samples [49,50,52]. Against this backdrop, the DSS-CH should be understood as a foundation for mechanism- and development-sensitive research rather than as a definitive statement on the structure or causal status of digital stress.

### 5.1. Internal Structure (RQ1)

Model comparisons clearly rejected a strictly unidimensional representation under an independent-clusters CFA and supported a three-facet structure (Excessive Screen Time, Compulsive Media Behavior, Approval Anxiety). Allowing cross-loadings in ESEM yielded a more realistic representation of item covariance and reduced interfactor correlations, suggesting that strict zero cross-loading constraints may inflate associations between facets [43]. This pattern aligns with conceptual accounts portraying digital stress as multidimensional and comprising interrelated components such as availability stress, connection overload, approval anxiety, FoMO, and online vigilance [20], as well as with measurement work in adolescent and young-adult samples [16].

The excellent fit of the bifactor-ESEM model further suggests that children’s responses reflect both a general digital stress liability and facet-specific variance. This dovetails with meta-analytic evidence indicating moderate associations between digital stress components and psychosocial distress, with relatively few differences across components [42]. If distinctions between components are already partly blurred in older samples, a comparatively strong general factor in late childhood and early adolescence is theoretically plausible. Developmental research shows that younger respondents often exhibit less differentiated factor structures for complex psychosocial constructs, with differentiation increasing across adolescence [45,46].

At the same time, the three-facet solution can be interpreted as a developmentally tuned mapping onto existing frameworks rather than as a competing taxonomy. Compulsive Media Behavior may capture child-salient manifestations of constant availability, attentional capture, and vigilance; Approval Anxiety reflects evaluation-related pressures emphasized in adolescent models; and Excessive Screen Time represents appraisal-based strain related to perceived overuse and loss of control. This interpretation is consistent with VAR17_2 (“restless without device”), which may reflect affectively loaded discomfort when access is interrupted rather than availability pressure or norm-driven responding. In late childhood and early adolescence, such restlessness plausibly aligns with the appraisal-based self-discrepancy and loss-of-control strain captured by Excessive Screen Time. Such concrete and experience-near formulations are consistent with methodological recommendations for child self-report, which emphasize clarity and reduced cognitive burden [53].

### 5.2. Reliability and Facet Distinguishability (RQ2)

Reliability estimates were satisfactory across subscales, indicating that a brief and developmentally appropriate instrument can achieve acceptable score precision while retaining theoretically meaningful differentiation. This is notable given that most existing digital stress measures were developed for adolescents and adults and often contain more differentiated facets and greater item length [16].

Moderate intercorrelations among subscales are consistent with the portrayal of digital stress as multifaceted yet interconnected [17,20]. The reduction in factor correlations under ESEM compared to CFA further suggests that some overlap in traditional CFA solutions may be methodological rather than substantive [43]. Substantively, this supports the practice of reporting facet profiles in addition to (or instead of) a total score, as different dimensions may imply distinct mechanisms and intervention targets.

A notable descriptive finding was the pronounced floor tendency for Approval Anxiety. Developmental accounts suggest that public self-presentation and feedback sensitivity become increasingly salient across adolescence as peer evaluation processes intensify [51]. In late childhood and early adolescence, digital environments and feedback ecologies are more heterogeneous and often constrained, which may explain lower average endorsement. Approval anxiety may therefore function as an emergent or subgroup-specific vulnerability marker at this age, warranting further investigation of moderation by platform access, activity type, and peer context.

### 5.3. Measurement Invariance (RQ3)

Evidence for scalar invariance across gender (girls vs. boys) and across younger (10–11) versus older (12–14) children supports the interpretability of latent mean comparisons. This is particularly important given that heterogeneity by person and developmental timing is a central theme in the digital media literature [49,54]. Without invariance, apparent group differences could reflect differential item interpretation or response thresholds rather than substantive differences in digital stress.

Establishing scalar invariance enables future research to examine whether gender- and age-related differences in digital stress levels or facet profiles emerge in childhood, without immediate concerns about measurement bias. This is relevant given mixed findings in adolescent research, where some studies report gender differences in digital stress frequency or related processes, while meta-analytic syntheses suggest limited moderation by sex and age [42]. As expected, the latent mean comparisons suggest higher levels across all three facets in older children, with the largest difference for approval anxiety. Gender differences were concentrated in approval anxiety (and to a lesser extent excessive screen time), whereas compulsive media behavior showed no reliable difference.

### 5.4. Implications for Future Research

While the present findings support the DSS-CH as a psychometrically credible starting point, measurement progress must be followed by stronger research designs to address the current predominance of cross-sectional and observational evidence [49,50,51]. Key priorities include:

1. External and predictive validity: Examining associations between DSS-CH facets and theoretically relevant outcomes (e.g., psychosocial distress, sleep, school functioning), and testing incremental validity beyond general negative affect or overall media engagement [16,20].
2. Longitudinal and within-person designs: Evaluating temporal stability, longitudinal measurement invariance, and reciprocal relations between digital stress and psychosocial outcomes, ideally using within-person approaches that better align with mechanism-based claims [49,52].
3. Affordance- and activity-specific integration: Linking DSS-CH scores to concrete digital affordances and activity patterns (e.g., notification frequency, public visibility contexts), in line with calls to move beyond monolithic “screen time” explanations [51].
4. Multi-method approaches: Combining self-report with diary/EMA designs and, where ethically feasible, behavioral indicators to reduce common-method bias and better capture real-time appraisal processes.

Importantly, the DSS-CH provides researchers, educators, and parents with a practical tool to assess children’s subjective experiences of digital stress, identify at-risk individuals, and inform evidence-based interventions. By clarifying how digital stress manifests in late childhood and early adolescence, this instrument enables more targeted studies on the mechanisms linking digital media use to well-being, ultimately supporting healthier technology engagement in everyday life.

Taken together, the present findings contribute to clarifying the internal structure and measurement properties of digital stress in late childhood and early adolescence. By providing a developmentally appropriate instrument with demonstrated reliability and invariance, this work lays the groundwork for more precise, mechanism-oriented, and longitudinal investigations of how digital stress unfolds across developmental stages and interacts with children’s coping resources and psychosocial adjustment.

## Figures and Tables

**Table 1:** Scale descriptives and reliability.

Factor	Items	Example Item	M	SD	Skew	Kurtosis	α	CR	AVE
Excessive screen time (EST)	3	I spend more time on digital devices than I would like.	1.93	0.75	0.55	−0.39	0.85	0.85	0.65
Compulsive media behavior (CMB)	3	I feel stressed or overwhelmed because I am constantly bombarded with information and messages.	1.56	0.64	1.32	1.38	0.81	0.83	0.59
Approval anxiety (AAN)	4	I am afraid that my posts (e.g., Reels, photos, Stories) will not be well received.	1.24	0.49	2.58	7.16	0.89	0.90	0.70

**Table 2:** Associations among subscales.

Pair	r (Pearson)	95% CI
EST—CMB	0.56	[0.489, 0.630]
EST—AAN	0.33	[0.260, 0.4064]
CMB—AAN	0.47	[0.397, 0.537]

**Table 3:** Factor correlations: CFA vs. ESEM.

Model	r (EST, CMB)	r (EST, AAN)	r (CMB, AAN)
CFA 3-factor	0.69	0.44	0.60
ESEM 3-factor	0.43	0.44	0.42

*Note*: all coefficients (*p* < 0.001), standardized coefficients.

**Table 4:** Goodness-of-fit indices of different measurement approaches.

Model	χ^2^	df	CFI	TLI	RMSEA [90% CI]	SRMR
CFA 1-factor	253.31 ***	35	0.810	0.757	0.146 [0.137, 0.155]	0.093
CFA 2-factor (overuse + compulsion vs. approval)	135.08 ***	34	0.906	0.873	0.099 [0.089, 0.108]	0.068
CFA 2-factor (excessive time vs. psychosocial pressure)	169.72 ***	34	0.887	0.850	0.108 [0.098, 0.117]	0.075
CFA 3-factor (EST, CMB, AAN)	73.25 ***	32	0.958	0.940	0.068 [0.058, 0.078]	0.057
ESEM 3-factor	9.46 ***	18	0.999	0.998	0.013 [0.000, 0.034]	0.012
Bifactor-ESEM (G + 3 specifics)	8.99 ***	15	0.999	0.998	0.012 [0.000, 0.038]	0.012

*Note*: *** χ^2^-test significant (*p* < 0.001).

**Table 5:** Standardized loadings (STDYX): ESEM and bifactor-ESEM.

Item	Paraphrase	Intended	ESEM EST	ESEM CMB	ESEM AAN	Bifactor G	Bifactor EST	Bifactor CMB	Bifactor AAN
VAR17_1	More time than intended	EST	0.81 ***	−0.07	0.05	0.50 ***	0.71 ***	0.04	0.14
VAR17_3	Hard to control time	EST	0.86 ***	−0.11	0.02	0.59 ***	0.56 ***	−0.09	−0.00
VAR19_3	Online longer than planned	EST	0.76 ***	0.03	0.02	0.60 ***	0.49 ***	0.01	−0.00
VAR17_2	Restless without a device	CMB	0.48 ***	0.20 ***	0.17 **	0.70 ***	0.20	**0.06**	0.01
VAR17_5	Always reachable	CMB	0.20 ***	0.66 ***	0.13 *	0.67 ***	0.09	0.45 **	0.07
VAR17_6	Reply immediately	CMB	0.02	0.86 ***	0.01	0.54 ***	0.01	0.81 ***	0.00
VAR19_7	Worry posts are not liked	AAN	−0.08 *	0.13 **	0.85 ***	0.62 ***	−0.06	0.09	0.63 ***
VAR19_8	Restless after posting	AAN	−0.04	−0.01	0.94 ***	0.59 ***	0.02	0.03	0.75 ***
VAR19_9	Insecure before posting	AAN	−0.01	−0.12 **	0.92 ***	0.54 ***	0.03	−0.07	0.72 ***
VAR19_10	Stressed if a few likes	AAN	0.17 **	0.01	0.71 ***	0.71 ***	0.01	−0.06	0.46 *

*Note*: Bold = targeted loadings; * *p* < 0.05, ** *p* < 0.01, *** *p* < 0.001.

**Table 6:** Measurement Invariance.

Grouping Variable	Model	χ^2^	df	*p*	CFI	RMSEA [90% CI]	Δ χ^2^	df	*p*
Gender	Configural	191.98	64	<0.001	0.973	0.066 [0.056, 0.077]	-	-	-
	Scalar	202.25	88	<0.001	0.976	0.054 [0.044, 0.063]	19.97	24	0.698
Age Group	Configural	208.09	64	<0.001	0.976	0.071 [0.060, 0.082]	-	-	-
	Scalar	232.32	88	<0.001	0.976	0.060 [0.051, 0.070]	33.69	24	0.090

*Note*: CFI = Comparative Fit Index; RMSEA = Root Mean Square Error of Approximation; CI = confidence interval; Δ = change.

## Data Availability

The data presented in this study are available on request from the corresponding author due to privacy and ethical restrictions.
